# The Role of District Wellness Policies in Encouraging Student Participation in the School Breakfast Program, United States

**DOI:** 10.3390/nu12082187

**Published:** 2020-07-23

**Authors:** Julien Leider, Wanting Lin, Elizabeth Piekarz-Porter, Lindsey Turner, Jamie F. Chriqui

**Affiliations:** 1 Institute for Health Research and Policy, University of Illinois Chicago, 1747 W. Roosevelt Road, M/C 275, Chicago, IL 60608, USA; wlin33@uic.edu (W.L.); epiekarz@uic.edu (E.P.-P.); jchriqui@uic.edu (J.F.C.); 2 Division of Health Policy and Administration, School of Public Health, University of Illinois Chicago, 1603 W. Taylor Street, M/C 923, Chicago, IL 60612, USA; 3 College of Education, Boise State University, 1910 University Drive, Boise, ID 83725, USA; lindseyturner1@boisestate.edu

**Keywords:** child nutrition, School Breakfast Program, district wellness policy, legal epidemiology, policy surveillance

## Abstract

Eating breakfast is associated with better academic performance and nutrition and lower risk of obesity, but skipping breakfast is common among children and adolescents, and participation in the U.S. Department of Agriculture’s School Breakfast Program (SBP) is low. This study assessed the association between school district wellness policy provisions coded as part of the National Wellness Policy Study and student SBP participation and acceptance of the breakfasts provided using cross-sectional survey data from the School Nutrition and Meal Cost Study. Separate survey-adjusted multivariable logistic regressions were computed, linking students eating (*N* = 1575) and liking (*N* = 726) the school breakfast to corresponding district policy measures, controlling for school and student characteristics. Strong district policy, as opposed to no policy, was associated with significantly higher odds of students eating the school breakfast (odds ratio (OR): 1.86; 95% CI: 1.09, 3.16; *p* = 0.022), corresponding to an adjusted prevalence of 28.4% versus 19.2%, and liking the school breakfast (OR: 2.14; 95% CI: 1.26, 3.63; *p* = 0.005), corresponding to an adjusted prevalence of 69.0% versus 53.9%. District policy has the potential to play an important role in encouraging higher levels of SBP participation.

## 1. Introduction

While skipping breakfast is common among children and adolescents, eating breakfast is associated with better academic performance, improved nutrition, and lower risk of obesity [1,2,3,4,5]. Concerningly, skipping breakfast is particularly prevalent among female and older children and adolescents, those of lower socioeconomic status, and those from urban as opposed to suburban or rural areas; there is also mixed evidence for higher prevalence of breakfast skipping among non-Hispanic black and Hispanic youth compared to non-Hispanic white youth [1,3,6,7,8,9]. The School Breakfast Program (SBP), administered by the U.S. Department of Agriculture (USDA), provides breakfasts that are required to meet federal nutrition standards and that are provided at free- or reduced-price for lower-income students [10]. In fiscal year 2019, 14.77 million children participated in the SBP each day, on average [11]. Past studies have found that availability of the SBP is associated with lower likelihood of breakfast skipping, particularly for children from low-income families [12], that state policies requiring schools to provide the SBP are associated with reduced food insecurity for young children [13], and that availability of the SBP may eliminate differences between food-secure and food-insecure children in eating breakfast [14]. Availability and consumption of the SBP have been found to be associated with improved nutritional outcomes [15,16,17,18], and consumption of the SBP has been found to be associated with lower body mass index (BMI) and risk of obesity, after accounting for selection bias [19,20,21], although there is some mixed evidence on this [22]. Furthermore, availability of the SBP has been linked to increased attendance and cognitive achievement in math, reading, and science [23,24].

Although an estimated 94% of public, non-charter schools that participate in the National School Lunch Program (NSLP) also participate in the SBP [25], student-level participation in the SBP has been low relative to the NSLP throughout the program’s history [26]. In fiscal year 2019, the number of children participating in the SBP was nearly half of the 29.6 million who participated in the NSLP [27]. Even among children eligible for free or reduced-price meals, the number participating in the SBP (12.54 million) was only over half the number participating in the NSLP (21.8 million) [11,27]. This is particularly troubling given that only 58% of eligible children participated in the NSLP in fiscal year 2017 (the most recent year for which data were available) [28]. This low participation in the SBP is notable given that income eligibility determining whether students were eligible to receive meals for free or at a reduced price was the same for both programs, and similar numbers of schools participated in both programs in the 2018–2019 school year (90,587 for the SBP and 96,781 for the NSLP [26]). Participation in the SBP is higher among those eligible for free or reduced-price meals, those of lower socioeconomic status, those in elementary as compared to middle and high school, male youth, those who are not non-Hispanic white, and those from urban compared to suburban and rural areas [6,29,30,31,32,33]. Studies have found that logistical factors, including lack of time in the morning or difficulty with bus schedules needed for children to arrive at the school cafeteria in time for breakfast, are a primary barrier to eating the school breakfast [34,35,36,37,38,39]. Social stigma associated with participating in the program, based on beliefs that it signals a student’s lower-income status or lack of parental investment, also plays a significant role [34,35,36]. Students and parents also cite concerns about the quality of the food offered in explaining lack of participation in the SBP, with students expressing a desire to be involved in taste tests and planning the menu, and parents wishing to be better informed as to the menu [34,35,36,37,38,39].

Several strategies are available to schools to overcome these barriers and increase participation in the SBP. Providing breakfast in the classroom has been associated with increased participation [3,22,31,40,41,42,43,44,45], as well as better school attendance [40] and better academic performance [46]. Offering a grab-and-go breakfast and second chance breakfasts are other options recommended by the USDA [47] that have also been associated with increased participation [42,44,48,49,50,51]. Other options include expanding cafeteria hours, mobile food carts, mandatory time in the cafeteria, and using marketing campaigns to engage students [31,39,43,45,50].

In the United States, school districts that participate in any federal child nutrition programs, including the SBP and NSLP, are required to have a wellness policy in place to address nutrition and physical activity goals for students [52,53,54,55]. As part of the policy, districts are required to include “standards and nutrition guidelines for all foods and beverages sold to students during the school day on each participating school campus” that are consistent with the meal requirements for the NSLP and SBP [55]. Additionally, prior research has documented that school nutrition-related practices are greater in districts with policies related to those practices [56,57,58,59]. Thus, given the district policy mandate that wellness policies include standards and guidelines that meet the SBP standards, district policies offer a potential mechanism to ensure the availability of the SBP in schools and to incorporate strategies to increase participation in the SBP by schools and students.

To the authors’ knowledge, this study is the first to analyze the nationwide association between U.S. school districts’ policies related to school breakfast and students’ (1) eating and (2) liking school breakfast. This study aimed to expand the field’s understanding of the factors that influence student participation in the SBP and test the extent to which district policy can promote student participation in the SBP and acceptance of the breakfasts provided.

## 2. Materials and Methods

### 2.1. Data and Design

This was a cross-sectional study. Data on student participation in and liking of the school breakfast were obtained from the School Nutrition and Meal Cost Study (SNMCS), which was conducted during the 2014–2015 school year on behalf of the USDA Food and Nutrition Service [60]. This study relied on de-identified data provided by Mathematica Policy Research after linking our policy variables. Students were randomly sampled from lists of students enrolled at sampled schools and were interviewed in person. The survey-weighted data from these interviews are nationally representative of students enrolled in public, non-charter schools participating in the NSLP. This study was deemed to “not involve human subjects” by the University of Illinois Chicago Institutional Review Board (protocol #2020-0448).

SNMCS data were linked to National Wellness Policy Study data on school district wellness policies for each district where the students were enrolled in school. Details on the district policy collection are provided elsewhere [52]. Briefly, policies were collected through internet research with telephone follow-up to verify complete collection. To ensure that the policies were in place at the time of the SNMCS survey fielding (2014–2015 school year), we compiled policies that were effective as of the day after Labor Day 2014 (2 September 2014; this was a proxy for the beginning of the school year).

### 2.2. Measures

#### 2.2.1. Eating/Liking School Breakfast

Student participation in the SBP on a given target day was an SNMCS measure derived primarily from administrative data [60]. For these analyses, this measure was set to missing for students in schools that did not serve breakfast, as they did not have the option of participating in the SBP. Students’ opinions on school breakfasts were obtained from a question on the Child/Youth Interview, which included a range of questions regarding school breakfast and lunch, as well as physical activity and height and weight measurements. The question of interest asked students, “What do you think about school breakfast? Do you like it, think it is only okay, or don’t like it?” This question was only asked of students in schools that served breakfast who indicated that they ate the school breakfast at least sometimes. This question was also skipped for students who responded, “food is good” in response to another question asking, “What is the number one reason you eat school breakfasts?” Those cases were recoded to “like it” for the question about liking school breakfast. For purposes of analyses, responses to this question were dichotomized as “like it” versus “only okay” or “don’t like it” due to the low prevalence of “don’t like it” as a response.

#### 2.2.2. District Policy

All district policies were double-coded using a detailed ordinal coding tool [61] that built off previously proven reliable and valid district policy coding tools [62,63]. For this analysis, two district policy variables were used: (1) whether policies address access to and/or promotion of the SBP, and (2) whether policies specify strategies to increase participation in school meal programs (which was coded for school meal programs generally and was not specific to breakfast). Specific strategies to increase participation included promotional mailings, altered bus schedules, student input on the menu, “grab and go”, and other alternative breakfast serving options. Each variable was coded on the basis of whether there was no policy on the topic, only a weak policy that mentioned or encouraged the topic, or a strong policy with a specific or definitive requirement.

#### 2.2.3. Control Variables

Analyses controlled for school and student characteristics using data from the SNMCS and the National Center for Education Statistics [64]. The student racial/ethnic distribution at the school level was grouped into four categories previously used in the literature [65]: ≥66% white, ≥50% black, ≥50% Hispanic, or other. The school percentage of students eligible for free/reduced-price lunch (FRPL) was categorized as ≤37.42%, >37.42–63.37%, or >63.37%, on the basis of tertiles for the overall SNMCS school sample. School urbanicity was categorized as urban, suburban, or rural. School size was categorized as small (fewer than 500 students), medium (500 to 999 students), or large (1000 or more students). Analyses also controlled for whether the school served breakfast free of charge to all students. Region was classified on the basis of Census definitions as West, Midwest, South, or Northeast [66]. Student grade ranged from 1–12 and was treated as a continuous measure in analyses. Student race/ethnicity was categorized as non-Hispanic white, non-Hispanic black, Hispanic, or other (including multiracial). Student household income as a percentage of the poverty level was categorized as ≤130%, >130–185%, and >185%, on the basis of the thresholds used for determining FRPL eligibility. Analyses also controlled for student gender.

### 2.3. Study Sample

**Figure 1:**
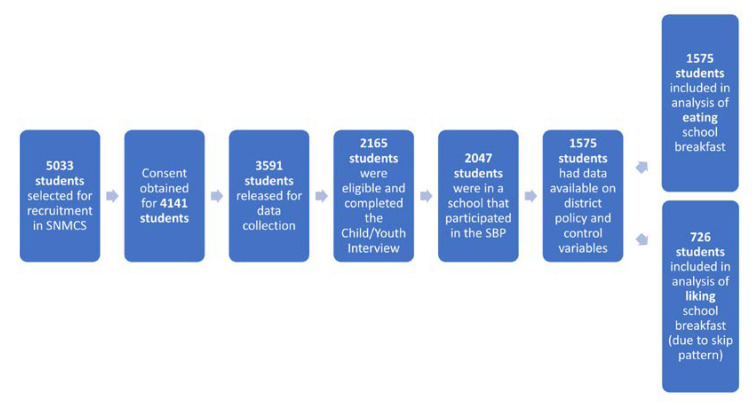
Analytical sample derivation.

Figure 1 shows the process by which the final analytical samples were derived. A total of 5033 students were selected for recruitment in SNMCS; consent was obtained for 4141, and 3591 were released for data collection. Of those released for data collection, 2165 were eligible and completed the Child/Youth Interview (63.6% weighted response rate [60]). Of the respondents, 2047 students were in schools that participated in the SBP. Missing data on control variables left 1580 students, while missing district policies left 1575 students. This was the sample size for the analysis of eating school breakfast. The analysis of liking school breakfast was limited to students who indicated they ate the school breakfast at least sometimes (*n* = 726 students). Statistics shown herein may differ from those in the SNMCS Final Report [60] due to differences in the analytical sample and the particular measures used.

### 2.4. Data Analysis

Descriptive statistics were computed separately for the two analyses, given the smaller sample of students who ate the school breakfast at least sometimes and therefore answered the question on liking school breakfast. Separate multivariable logistic regression analyses were computed linking students’ eating and liking school breakfast to corresponding district policy measures, controlling for school and student characteristics. Adjusted prevalence estimates were computed on the basis of these models, showing average predicted probabilities of these outcome measures if students attended school in a district with no policy, a weak (encouraged) policy, or a strong (required) policy. Additional models adding interaction terms between district policy and school or student characteristics were run to test for moderation by district policy where there was a statistically significant association between the given characteristic and the outcome. To limit multiple testing, statistical significance for categorical variables was computed on the basis of a Wald test for the joint significance of all levels of the variable, and the presence of moderation was determined on the basis of a Wald test for the joint significance of all interaction terms. Statistical significance was determined at the *p* < 0.05 level on the basis of two-tailed tests [67]. All analyses took account of the survey design and weights, treating strata with a single sampling unit as certainty units in analyses, and were conducted in Stata/SE (version 15.1, StataCorp LP, College Station, TX, USA; 2016).

## 3. Results

### 3.1. Survey-Weighted Analytical Sample Characteristics

Table 1 shows survey-weighted characteristics of the analytical samples. Nearly a quarter (24.4%) of students ate the school breakfast on the target day. Most students who ate breakfast at school liked the school breakfast (56.8%), while 37.2% thought it was “only okay” and only 6.1% did not like it. Most students (51.9%) in the analysis of eating school breakfast were in a district with a strong policy on the SBP, while only 17.7% of students in the analysis of liking school breakfast were in a district with a strong policy on strategies to increase participation in school meal programs.

About a quarter (26.8%) of students in the analysis of eating school breakfast were in a school that served breakfast free of charge to all students, while that was true for nearly two-fifths (39.6%) of students in the analysis of liking school breakfast. Students were in schools with a range of racial/ethnic compositions. For the analysis of eating school breakfast, students were about evenly distributed among school-level tertiles of FRPL eligibility, while more than half (53.1%) were in schools with a high percentage of students eligible for FRPL for the analysis of liking school breakfast. Between roughly two-fifths and one-half of students in both analyses were in suburban schools and schools with 500–999 students, and nearly half of students were in the South.

Students in both analyses were distributed across all 12 grade levels, with about half being female. In the analysis of eating school breakfast, nearly half (49.5%) of students were non-Hispanic white, with about one-quarter (27.7%) being Hispanic, and 14.5% being non-Hispanic black; these percentages were 38.1%, 33.5%, and 20.8%, respectively, for the analysis of liking school breakfast. Fewer than 10% of students in either analysis fell into the “other” race/ethnicity category. For the analysis of eating school breakfast, more than half (50.6%) of students were in a household with income above 185% of the poverty level, with 38.8% in a household with income of 130% of the poverty level or less. These percentages were reversed for the analysis of liking school breakfast, with 56.6% of students in a household with income of ≤130% of the poverty level and 30.2% of students in a household with income >185% of the poverty level.

### 3.2. The Association between District Policy on the School Breakfast Program and Students’ Eating School Breakfast

Table 2 shows the results of a logistic regression model linking district policy on the SBP to students’ eating school breakfast. Having a strong district policy as opposed to no policy was associated with significantly higher odds of eating the school breakfast (odds ratio (OR): 1.86; 95% CI: 1.09, 3.16; *p*-value = 0.022), corresponding to an adjusted prevalence of 28.4% versus 19.2%. Having a weak policy as opposed to no policy was not significantly associated with the odds of eating the school breakfast (OR: 1.26; 95% CI: 0.68, 2.34; *p*-value = 0.453).

Students in schools that served breakfast free of charge to all students were more likely to eat the school breakfast (OR: 3.52; 95% CI: 2.18, 5.69; *p*-value < 0.001). Household income was also associated with this outcome (*p*-value < 0.001), with students with household income ≤130% (OR: 1.95; 95% CI: 1.33, 2.86) and >130–185% (OR: 2.87; 95% CI: 1.94, 4.25) of the poverty level more likely to eat the school breakfast than those with household income >185% of the poverty level. Similarly, student race/ethnicity (*p*-value = 0.041) was associated with eating school breakfast; specifically, non-Hispanic black (OR: 2.29; 95% CI: 1.21, 4.33) and Hispanic (OR: 1.80; 95% CI: 1.06, 3.07) students were more likely to eat the school breakfast than non-Hispanic white students. School-level racial/ethnic composition (*p*-value = 0.026) was also associated with eating school breakfast, with students in schools with ≥50% black students (OR: 0.39; 95% CI: 0.17, 0.91) and those with ≥50% Hispanic students (OR: 0.43; 95% CI: 0.21, 0.90) being less likely to eat the school breakfast than students in schools with ≥66% white students. Finally, school size (*p*-value = 0.005) was associated with this outcome; specifically, students in small schools were more likely than those in large schools to eat the school breakfast (OR: 2.52; 95% CI: 1.37, 4.65).

None of the observed associations with school or student characteristics were moderated by district policy, except for school size (*p*-value = 0.031). Strong district policy was specifically associated with students eating school breakfast for small schools (OR: 3.89; 95% CI: 1.70, 8.92) and not schools of other sizes; for small schools only, weak district policy was also associated with students eating school breakfast (OR: 3.29; 95% CI: 1.56, 6.95).

### 3.3. The Association between District Policy on Strategies to Increase Participation in School Meal Programs and Students’ Liking School Breakfast

Table 3 shows the results of a logistic regression model linking district policy on strategies to increase participation in school meal programs to students’ liking school breakfast. Having a strong district policy as opposed to no policy was associated with significantly higher odds of liking the school breakfast (OR: 2.14; 95% CI: 1.26, 3.63; *p*-value = 0.005), corresponding to an adjusted prevalence of 69.0% versus 53.9%, while having a weak district policy was not significantly associated with the odds of liking the school breakfast (OR: 1.02; 95% CI: 0.61, 1.71; *p*-value = 0.926).

Students in schools that served breakfast free of charge to all students were less likely to like the school breakfast (OR: 0.57; 95% CI: 0.34, 0.94; *p*-value = 0.028). School urbanicity was associated with liking school breakfast (*p*-value = 0.017), with students in suburban schools having the highest odds of liking the school breakfast, followed by urban and then rural students; pairwise comparisons with urban schools were not statistically significant, but the difference between suburban and rural schools was (*p*-value = 0.005; not shown in tables). School size was also associated with liking school breakfast (*p*-value = 0.027), with students in small (OR: 0.41; 95% CI: 0.18, 0.91) and medium-size (OR: 0.36; 95% CI: 0.17, 0.75) schools having lower odds of liking the school breakfast than those in large schools. Finally, students in higher grades were less likely to like the school breakfast.

Associations were not moderated by district policy, except in the case of school size (*p*-value = 0.041). Strong district policies were specifically associated with liking school breakfast in small (OR: 3.10; 95% CI: 1.14, 8.46) and medium-size (OR: 2.51; 95% CI: 1.47, 4.29) schools and not large schools.

## 4. Discussion

This study found that strong (i.e., required) district policies on the SBP and strategies to increase participation in school meal programs were positively associated with students’ eating and liking school breakfast, respectively. This represents an important policy opportunity, given that levels of student participation in the SBP are generally low [26], and studies show the association of SBP availability/consumption with important outcomes, including lower likelihood of breakfast skipping [12], improved nutritional outcomes [15,16,17,18], lowered BMI and risk of obesity [19,20,21], and increased attendance and cognitive achievement [23,24].

Participation in the SBP is much lower than participation in the NSLP despite similar income eligibility and numbers of schools participating in both programs [25,26], but there are a number of avenues available to encourage SBP participation. Logistical factors such as difficulties arriving in time for breakfast are a primary barrier to SBP participation [34,35,36,37,38,39]. Providing breakfast in the classroom, offering a grab-and-go or second chance breakfast, expanding cafeteria hours, and using mobile food carts to increase the number of locations where students can access breakfast are all options that can make it easier for students to eat the school breakfast and have been shown to be associated with increased participation [3,22,31,40,41,42,43,44,45,48,49,50]. Concerns about food quality are also an important factor in SBP participation [34,35,36,37,38,39] that can be addressed through marketing campaigns to engage students, as well as activities such as taste tests [39,50]. Stigma associated with SBP participation is also a major barrier [34,35,36] that could potentially be addressed through these measures by encouraging participation by a broader range of students. Strong district policies promoting the SBP and strategies to increase participation in school meals could help ensure best practices are used to promote SBP participation.

This study is unique in analyzing district policy associations using nationwide data, but results are consistent with past studies of school-level interventions showing positive associations with SBP participation [3,31,39,40,41,42,43,44,45,48,49,50]. This study also found that strong district policies with specific requirements related to the SBP and/or related to strategies to increase student participation in school meals—but not weak district policies that only mentioned or encouraged the SBP and/or strategies—were associated with students’ eating and liking the school breakfast. This is consistent with past studies of district wellness policy provisions where significant associations with outcomes were seen for strong policies [57,68], and emphasizes the importance of including specific, enforceable language in district policy.

Similar to previous studies, this study found disparities in SBP participation by race and income [29,30,31,33]. This study also found higher participation when breakfast is served free of charge to all students, and in small compared to large schools. Disparities in liking school breakfast, including students being less likely to like the school breakfast in rural compared to suburban schools and in small and medium-size schools compared to large schools, as well as lower odds of liking the school breakfast among students in higher grade levels, suggest opportunities to increase participation by making the school breakfast more palatable to students. It is also noteworthy that students were less likely to like the school breakfast in schools where breakfast was served free of charge to all students. This suggests lower quality breakfasts may be served in these schools and thus represents an important area for future research. Except in the case of school size, disparities were not moderated by district policy, suggesting further work may be needed to bridge these divides.

The findings of this study should be considered in the context of the following limitations. First, because this was a cross-sectional study utilizing only one year of data, only associations with district policies can be established, not causal relationships. While analyses controlled for several variables likely to influence students’ eating and liking school breakfast, such as breakfast being served free of charge to all students, the school-level percentage of FRPL-eligible students, school- and student-level race/ethnicity, and student-level household income, future studies should use longitudinal data to account for unobserved confounders such as community attitudes favoring eating breakfast at home. Second, we could not control for student BMI or other student weight-related outcomes because we would have lost too much statistical power due to missing data. Future studies should control for this. Third, district policies were not coded as to whether they encouraged or required universal free breakfast and so we were unable to examine that as a predictor, although such policies could play an important role in reducing stigma and encouraging SBP participation, as we do find a positive association of offering universal free breakfast with students eating the school breakfast, despite finding a negative association with students liking the school breakfast. Fourth, this study specifically sought to provide evidence on whether district policies could promote SBP participation, and as such, did not examine student dietary or academic outcomes. Finally, this study relied on self-reported survey data which may be subject to error.

## 5. Conclusions

Using nationwide data, this study found that strong district policies on the SBP and strategies to increase participation in school meal programs were positively associated with students’ eating and liking school breakfast. This study used the best available nationally representative data, collected on behalf of the USDA and covering 46 states and the District of Columbia and all grade levels, and employed multivariable models controlling for school and student sociodemographic and socioeconomic characteristics. Future research should explore this relationship with longitudinal data and examine the role of district policies on whether schools provide universal free breakfast. In addition, exploring the potential of policies to increase school food service programs’ use of breakfast promotion strategies such as second-chance breakfast or breakfast in the classroom would be important. The current study suggests that federally mandated district wellness policies provide an important opportunity to include requirements to promote SBP participation. A variety of strategies are available to promote SBP participation and can be written into district policies, including breakfast in the classroom, grab-and-go and second chance breakfasts, expanded cafeteria hours, and marketing campaigns. This study’s results are also likely to be relevant in other countries with nationwide school meal programs and local implementation.

## Figures and Tables

**Table 1:** Survey-weighted characteristics of analytical samples of students from the School Nutrition and Meal Cost Study.

	Analysis: Eating School Breakfast	Analysis: Liking School Breakfast
Variable	% (95% CI)	% (95% CI)
Outcome measures		
Student ate school breakfast on target day	24.4 (20.0–29.5)	
Student opinion of school breakfast		
Like it		56.8 (51.2–62.1)
Only okay		37.2 (31.7–43.1)
Don’t like it		6.1 (4.1–8.9)
Policy measures		
District policy on School Breakfast Program		
No policy or provision	26.5 (16.7–39.4)	
Weak policy	21.5 (12.9–33.7)	
Strong policy	51.9 (39.8-63.8)	
District policy on strategies to increase participation in school meal programs		
No policy or provision		52.7 (39.8–65.3)
Weak policy		29.6 (18.9–43.1)
Strong policy		17.7 (10.5–28.2)
Control variables		
Breakfast served free of charge to all students		
No	73.2 (62.9–81.5)	60.4 (47.3–72.2)
Yes	26.8 (18.5–37.1)	39.6 (27.8–52.7)
School race/ethnicity		
≥66% White	43.2 (32.8–54.2)	35.5 (25.0–47.5)
≥50% Black	7.8 (3.5–16.6)	11.9 (5.0–25.7)
≥50% Hispanic	16.7 (10.6–25.1)	24.6 (15.7–36.3)
Other	32.3 (24.0–41.9)	28.0 (20.5–37.0)
School percentage of students eligible for free/reduced-price lunch		
Low (≤37.42%)	30.1 (21.8–40.0)	16.1 (10.6–23.7)
Medium (>37.42–63.37%)	32.2 (24.4–41.1)	30.8 (22.5–40.5)
High (>63.37%)	37.7 (28.9–47.5)	53.1 (42.3–63.7)
School urbanicity		
Urban	25.6 (17.4–36.0)	30.5 (19.7–44.0)
Suburban	46.9 (36.2–57.9)	42.4 (30.6–55.1)
Rural	27.5 (18.1–39.4)	27.2 (17.8–39.0)
School size		
Small (fewer than 500 students)	26.1 (19.0–34.8)	33.0 (22.8–45.2)
Medium (500 to 999 students)	44.1 (35.1–53.4)	50.7 (38.9–62.4)
Large (1000 or more students)	29.8 (23.1–37.6)	16.3 (11.6–22.6)
Region		
West	17.5 (10.3–28.2)	17.9 (10.5–28.9)
Midwest	24.9 (15.9–36.8)	23.3 (13.9–36.5)
South	45.6 (33.8–57.9)	47.7 (34.6–61.2)
Northeast	12.1 (6.1–22.6)	11.0 (5.1–22.2)
Student grade		
1	9.1 (7.1–11.7)	13.1 (9.7–17.6)
2	9.8 (8.0–12.0)	13.7 (10.7–17.4)
3	10.6 (8.5–13.1)	16.3 (13.0–20.2)
4	8.8 (6.9–11.2)	11.4 (8.7–14.8)
5	8.7 (7.0–10.6)	9.6 (7.1–12.8)
6	6.9 (5.2–9.2)	6.3 (3.9–10.0)
7	6.8 (5.0–9.0)	4.6 (3.3–6.3)
8	6.7 (5.3–8.5)	5.4 (3.8–7.6)
9	9.8 (7.5–12.8)	6.8 (4.8–9.7)
10	8.2 (6.3–10.5)	5.0 (3.5–7.0)
11	8.8 (6.7–11.5)	3.8 (2.4–5.9)
12	5.8 (4.3–7.8)	4.0 (2.4–6.7)
Student gender		
Male	49.0 (46.0–52.0)	53.1 (48.5–57.6)
Female	51.0 (48.0–54.0)	46.9 (42.4–51.5)
Student race/ethnicity		
White, non-Hispanic	49.5 (42.1–56.8)	38.1 (29.5–47.6)
Black, non-Hispanic	14.5 (9.5–21.6)	20.8 (13.1–31.5)
Hispanic	27.7 (22.0–34.3)	33.5 (25.4–42.8)
Other (includes multi-racial)	8.3 (6.6–10.4)	7.5 (5.4–10.4)
Household income as a percentage of poverty level		
≤130%	38.8 (33.6–44.3)	56.6 (50.6–62.4)
>130–185%	10.5 (8.4–13.1)	13.3 (9.9–17.6)
>185%	50.6 (44.8–56.4)	30.2 (25.3–35.6)

CI, confidence interval. *n* = 1575 students for the eating school breakfast analysis and *n* = 726 students for the liking school breakfast analysis.

**Table 2:** Logistic regression results for the association between district policy on the School Breakfast Program and students’ eating of school breakfast *(n* = 1575 students).

Variable	OR (95% CI)	*p*-Value
District policy on School Breakfast Program		
No policy or provision	Referent	
Weak policy	1.26 (0.68–2.34)	0.453
Strong policy	1.86 (1.09–3.16)	0.022
Breakfast served free of charge to all students		
No	Referent	
Yes	3.52 (2.18–5.69)	<0.001
School race/ethnicity		
≥66% White	Referent	0.026
≥50% Black	0.39 (0.17–0.91)	
≥50% Hispanic	0.43 (0.21–0.90)	
Other	0.61 (0.37–1.02)	
School percentage of students eligible for free/reduced-price lunch		
Low (≤37.42%)	Referent	0.102
Medium (>37.42–63.37%)	1.73 (1.05–2.85)	
High (>63.37%)	1.53 (0.80–2.89)	
School urbanicity		
Urban	Referent	0.640
Suburban	0.78 (0.47–1.30)	
Rural	0.83 (0.47–1.49)	
School size		
Small (fewer than 500 students)	2.52 (1.37–4.65)	0.005
Medium (500 to 999 students)	1.48 (0.82–2.66)	
Large (1000 or more students)	Referent	
Region		
West	Referent	0.677
Midwest	0.71 (0.33–1.50)	
South	1.02 (0.53–1.95)	
Northeast	0.85 (0.39–1.89)	
Student grade (1–12)	0.96 (0.90–1.03)	0.233
Student gender		
Male	Referent	
Female	0.85 (0.58–1.25)	0.403
Student race/ethnicity		
White, non-Hispanic	Referent	0.041
Black, non-Hispanic	2.29 (1.21–4.33)	
Hispanic	1.80 (1.06–3.07)	
Other (includes multi-racial)	1.52 (0.75–3.06)	
Household income as a percentage of poverty level		
≤130%	1.95 (1.33–2.86)	<0.001
>130–185%	2.87 (1.94–4.25)	
>185%	Referent	
Adjusted prevalence (%) of students eating school breakfast		
No policy or provision	19.2	
Weak policy	22.4	
Strong policy	28.4	

CI, confidence interval; OR, odds ratio.

**Table 3:** Logistic regression results for the association between district policy on strategies to increase participation in school meal programs and students’ liking of school breakfast (*n* = 726 students).

Variable	OR (95% CI)	*p*-Value
District policy on strategies to increase participation in school meal programs		
No policy or provision	Referent	
Weak policy	1.02 (0.61–1.71)	0.926
Strong policy	2.14 (1.26–3.63)	0.005
Breakfast served free of charge to all students		
No	Referent	
Yes	0.57 (0.34–0.94)	0.028
School race/ethnicity		
≥66% White	Referent	0.117
≥50% Black	0.83 (0.37–1.85)	
≥50% Hispanic	0.68 (0.29–1.58)	
Other	0.48 (0.24–0.95)	
School percentage of students eligible for free/reduced-price lunch		
Low (≤37.42%)	Referent	0.211
Medium (>37.42–63.37%)	1.18 (0.59–2.37)	
High (>63.37%)	1.75 (0.82–3.71)	
School urbanicity		
Urban	Referent	0.017
Suburban	1.54 (0.88–2.67)	
Rural	0.78 (0.44–1.38)	
School size		
Small (fewer than 500 students)	0.41 (0.18–0.91)	0.027
Medium (500 to 999 students)	0.36 (0.17–0.75)	
Large (1000 or more students)	Referent	
Region		
West	Referent	0.403
Midwest	0.78 (0.41–1.49)	
South	1.01 (0.58–1.77)	
Northeast	0.59 (0.28–1.24)	
Student grade (1-12)	0.77 (0.71–0.83)	<0.001
Student gender		
Male	Referent	
Female	1.00 (0.62–1.60)	0.984
Student race/ethnicity		
White, non-Hispanic	Referent	0.103
Black, non-Hispanic	0.88 (0.49–1.58)	
Hispanic	1.16 (0.62–2.17)	
Other (includes multi-racial)	0.50 (0.22–1.12)	
Household income as a percentage of poverty level		
≤130%	0.98 (0.60–1.60)	0.589
>130–185%	0.76 (0.44–1.30)	
>185%	Referent	
Adjusted prevalence (%) of students liking school breakfast		
No policy or provision	53.9	
Weak policy	54.4	
Strong policy	69.0	

CI, confidence interval; OR, odds ratio.
